# Incidence and factors influencing the occurrence of postoperative thrombosis in patients with lower limb fractures across different age groups: a retrospective study

**DOI:** 10.1080/07853890.2025.2585602

**Published:** 2026-08-09

**Authors:** Mengwen Xue, Hui Liu, Rui An, Ruiping Bai, Li Zhang, Xin Shen

**Affiliations:** ^a^ Department of Anesthesiology, The First Affiliated Hospital of Xi’an Jiaotong University, Xi’an, China; ^b^ Biobank, The First Affiliated Hospital of Xi’an Jiaotong University, Xi’an, China; ^c^ Department of Anesthesiology, Honghui Affiliated Hospital of Xi’an Jiaotong University, Xi’an, China

**Keywords:** Postoperative venous thrombosis, lower limb fractures, young/middle-aged population, elderly population, predictive model

## Abstract

**Background:**

Postoperative venous thrombosis (PVT) is a common complication following lower limb fracture surgery, yet age-specific risk factors remain unclear. This study aimed to identify distinct predictors and develop tailored prediction models for young/middle-aged and elderly patients.

**Patients and methods:**

This multicentre retrospective cohort study analysed factors influencing of PVT in 483 patients undergoing lower limb fracture surgery at two tertiary hospitals between 1 January 2020 and 31 December 2022. Participants were divided into young/middle-aged (18–59 years, *n* = 269) and elderly (≥60 years, *n* = 214) groups for stratified analysis. Comprehensive perioperative variables were analysed. PVT was diagnosed via ultrasound within seven days postoperatively. Age-specific predictors were identified using multivariable logistic regression, and nomogram models were subsequently developed. Model performance was evaluated through the area under the curve (AUC).

**Results:**

The overall PVT incidence was 15.24% and 23.36% (*p* < 0.05) in young/middle-aged and elderly patients, respectively. Predictive of postoperative thrombosis among the young/middle aged population were intraoperative colloid infusion rate (OR = 0.784, 95% CI: 0.667–0.921), preoperative prothrombin time activity (OR = 0.947, 95% CI: 0.913–0.982) and postoperative anticoagulant use (OR = 7.117, 95% CI: 2.062–24.560) and among the elderly group were total intraoperative crystalloids (OR = 1.001, 95% CI: 1.000–1.001) and colloid infusion rate (OR = 1.101, 95% CI: 1.104–1.208), postoperative total protein (OR = 0.937, 95% CI: 0.886–0.990) and serum sodium (OR = 0.918, 95% CI: 0.848–0.994). The AUC and total score points for young/middle-aged group model was 0.760 and 204 points, and 0.750 and 116 points among the elderly.

**Conclusions:**

Fracture patients of different age groups require distinct risk management and perioperative treatments. This study developed age-specific PVT prediction models, highlighting the different risk profiles between young/middle-aged and elderly patients. For young/middle-aged patients, preoperative coagulation status should be optimized, postoperative anticoagulant overuse limited and fluid volume recovery enhanced. For elderly patients, intraoperative fluid input should be strictly restricted, and postoperative protein and electrolyte levels monitored.

## Introduction

Venous thromboembolism (VTE) is a well-recognized and potentially fatal complication following orthopaedic trauma [1]. Studies have shown that the incidence of postoperative venous thrombosis (PVT) in fracture patients is as high as 40% [2]. Among fracture patients, the formation of deep vein thrombosis (DVT) can be attributed to factors such as prolonged bed rest, post-traumatic inflammatory responses and blood stasis [3]. DVT is characterized by its high incidence, high disability rate and high mortality rate in perioperative patients. The incidence of DVT in patients with lower limb fractures ranges from 2.99% to 66.7% [4]. It also significantly impacts the patient’s recovery and prognosis [5,6].

Age is a recognized and unmodifiable risk factor for thrombosis [7]. Studies have found that although the age-standardized incidence of fractures has shown a declining trend over the past 30 years, the fracture incidence rate among the elderly population has been increasing [8]. Significant differences exist in the pathophysiological mechanisms and influencing factors of postoperative thrombosis between young/middle-aged patients and elderly patients, primarily manifested in vascular conditions, blood components, physical mobility, comorbidities and immune responses. However, there remains a gap in current research targeting different age groups [9], and existing studies primarily focus on preoperative conditions while lacking comprehensive consideration of patients’ entire perioperative period [9–13]. In fact, patient conditions develop in an integrated and continuous manner. Therefore, it is particularly important to conduct complete studies on the perioperative period of thrombosis complications following lower limb fracture surgery across different age groups and to identify relevant influencing factors.

This study aims to comprehensively assess the perioperative conditions of patients, precisely identify the incidence and influencing factors of thrombosis in patients with lower limb fractures across different age groups through stratified analysis, and respectively develop predictive models to guide the identification and early intervention of high-risk clinical populations.

## Patients and methods

### Study design, setting, period and ethical considerations

This study was conducted at the First Affiliated Hospital of Xi’an Jiaotong University and Xi’an Honghui Hospital, with approval from their Ethics Review Committees (Approval No. XJTUAF2024LSYY-263; 202406007). Both hospitals are representative and influential tertiary A-grade orthopaedic hospitals in Northwest China, characterized by large patient volumes and high medical standards. The clinical research was conducted in accordance with the Declaration of Helsinki. As a retrospective study, written informed consent was waived by both ethics committees. We retrospectively collected clinical data of patients who underwent lower limb fracture surgery between 1 January 2020 and 31 December 2022 at the two centres. Patients were stratified into groups based on postoperative thrombosis occurrence. The follow-up time for this study was seven days after the operation.

### Study population

#### A consecutive series of patients who met the inclusion criteria were enrolled in the study

*Inclusion criteria*: (1) Age ≥18 years; (2) patients who underwent surgery for closed lower limb fractures at the First Affiliated Hospital of Xi’an Jiaotong University and Xi’an Honghui Hospital between 1 January 2020 and 31 December 2022; (3) complete clinical medical records, including demographic data and perioperative documentation (e.g. diagnostic test results, therapeutic interventions and intraoperative management protocols); (4) no significant abnormalities in cardiac or other major organ functions; (5) American Society of Anesthesiologists (ASAs) classification of 1–3; (6) underwent ultrasonography before and after the operation.

*Exclusion criteria*: (1) Age <18 years; (2) missing critical information in clinical medical records; (3) no lower limb ultrasound before or after surgery; (4) presence of lower limb thrombosis before surgery; (5) presence of severe systemic diseases before surgery; (6) history of drug abuse, mental illness or alcoholism before surgery; (7) surgical duration of less than 120 min.

### Observation indicators

In this study, we conducted a statistical analysis of patients’ perioperative basic characteristics, medical history, comorbidities, various laboratory test results, surgery-related indicators and anaesthesia-related indicators. Specific variables are listed in Supplementary Table 1. The primary evaluation indicator was the incidence of PVT within seven days post-surgery. PVT was defined as the absence of preoperative lower limb venous thrombosis and the presence of new-onset thrombosis postoperatively, based on ultrasound results. According to the definition by the World Health Organization and China’s Law on the Protection of the Rights and Interests of the Elderly, individuals aged 60 years and above are defined as elderly.

All data in this study were extracted from the EMR-Q and Real-One systems, which are the primary data recording platforms at the research centre.

### Perioperative anticoagulation procedure

In accordance with the guidelines for perioperative VTE prevention in trauma orthopaedics, tailored anticoagulation protocols were implemented in this study based on individual patient profiles. These protocols included early mobilization, physical prophylaxis and anticoagulant therapy. The specific anticoagulant regimens utilized were: dalteparin sodium injection (50,000 IU once daily), heparin sodium injection (5000 IU once daily), enoxaparin sodium injection (60 mg once daily), nadroparin calcium injection (0.3 mL once daily) and low molecular weight heparin calcium injection (0.4 mL once daily).

### Statistical analysis

For sample size calculation, based on the 10EPV rule and our results, we needed >30 samples for the young/middle-aged group and >40 for the elderly group. All data analyses and the construction of the nomogram model were performed using SPSS 26.0 (SPSS Inc., Chicago, IL) and R 4.2.0 software (Vienna, Austria). Variables with a missing proportion greater than 3% were excluded, while those with less than 3% missing data were imputed using the mean or median of each variable. The sample size was estimated based on the 10 EPV (events per variable) rule, which ensures that each predictor parameter (each *β* coefficient in the regression equation) has at least 10 events when developing prediction models for binary or time-to-event outcomes.

Correlation analysis was conducted according to variable data types: the Pearson correlation coefficient and Spearman’s rank correlation were applied to continuous variables, Kendall’s tau to ordinal data, and the Phi coefficient to binary categorical variables.

Categorical data were assessed using the Chi-square test or Fisher’s exact test and presented as ratios. Continuous data, after normality and homogeneity of variance analyses, were expressed as mean ± standard deviation or median (interquartile range). The dependent variable was whether postoperative thrombosis occurred, and the independent variables were perioperative influencing factors. Perioperative indicators were analysed using univariate analysis and logistic regression analysis, with *p* < 0.05 considered statistically significant (Supplemental Table 1). The model’s discrimination was evaluated using the receiver operating characteristic (ROC) curve and the area under the ROC curve (AUC). An AUC of 0.5 indicates no discriminative ability, 0.7–0.9 indicates good discriminative ability, and greater than 0.9 indicates excellent discriminative ability.

Internal validation was performed using the bootstrap method with 500 resampling iterations to calculate the concordance index (*C*-index). When *C*-index >0.7 indicates good predictive performance of the model. The Brier score was used to assess prediction probability, with 0 representing perfect prediction and 1 indicating the worst prediction.

## Results

### Participant selection

**Figure 1.**
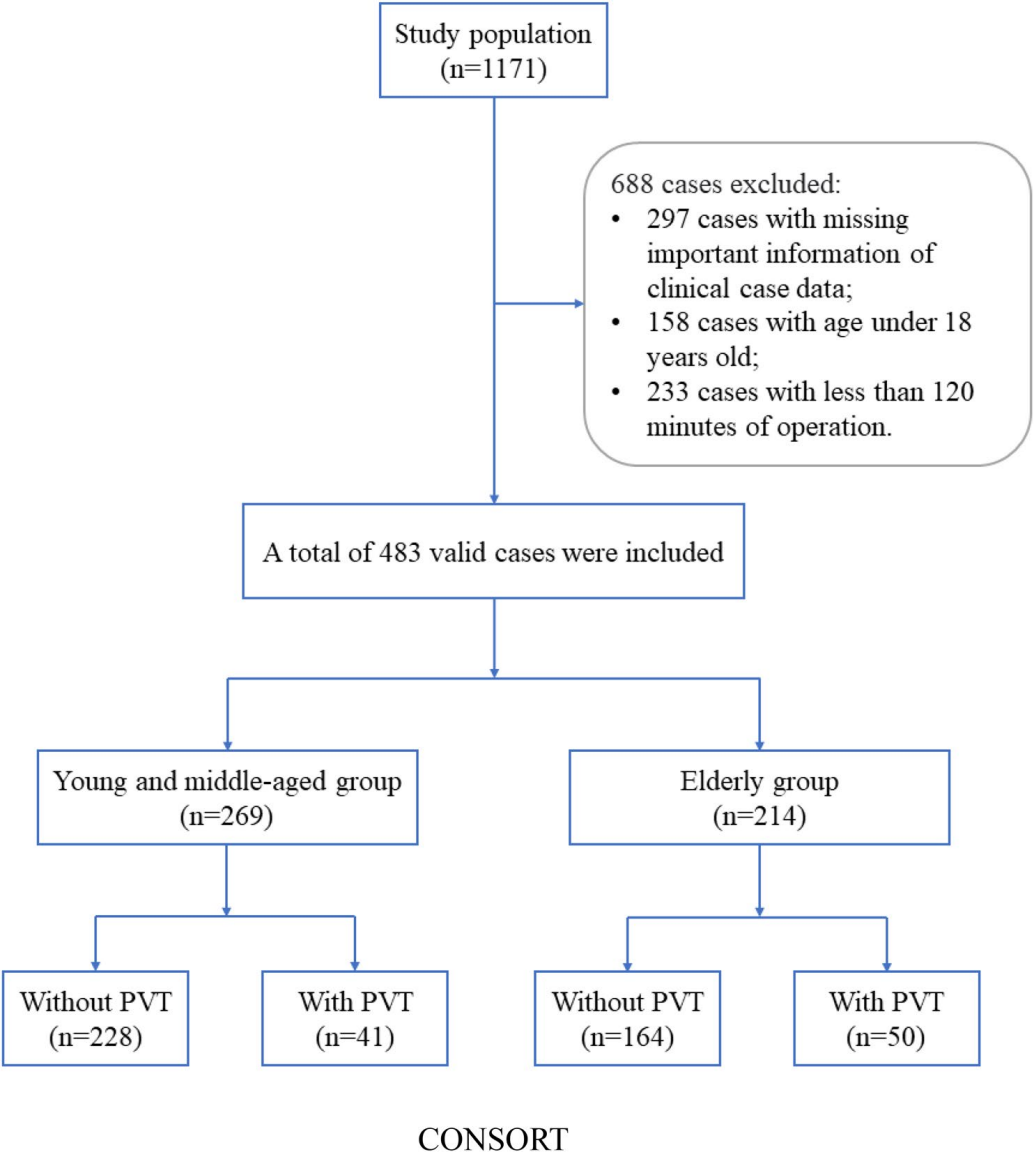
CONSORT.

During the study period, a total of 1171 patients underwent lower limb fracture surgery were initially enrolled from the two research centres. Among them, 297 patients were excluded due to missing critical clinical data, 158 patients were excluded for being under 18 years old, and 233 patients were excluded for surgical duration less than 120 min. Ultimately, 483 valid cases were included in the analysis (Figure 1).

### Participant characteristics of the study population

Among the cases included in this study, 269 cases (55.69%) were in the young/middle-aged population (ages 18–59), and 214 cases (44.31%) were in the elderly population (ages 60–95). Regarding fracture sites, femoral fractures had the highest incidence in this study, at 53.21%, followed by tibial and fibular fractures at 25.47%. In terms of surgical types, internal fixation was the most common, comprising 70.81%, followed by joint replacement at 27.74%, and external fixation at only 3.11% (Table 1).

**Table 1.** Incidence of thrombosis among patients with lower limb fractures at First Affiliated Hospital of Xi’an Jiaotong University and Xi’an Honghui Hospital, China, 2020–2022.

	Number (%)	Incidence of thrombosis, *N* (%)
Total population	483	91 (18.84)
Young and middle-aged group	269 (55.69)	41 (15.24)
Elderly group	214 (44.31)	50 (23.36)
Sex		
Male	262 (54.24)	48 (18.32)
Female	221 (45.76)	43 (19.46)
The site of the fracture		
Knee and patella	23 (4.76)	6 (26.09)
Hip and pelvis	19 (3.93)	3 (15.79)
Thighbone	257 (53.21)	32 (12.45)
Tibiofibula	123 (25.47)	10 (8.13)
Ankle	48 (9.94)	2 (4.17)
Others	54 (11.18)	4 (7.41)
Type of surgery		
External fixation	15 (3.11)	5 (33.33)
Joint replacement	134 (27.74)	26 (19.40)
Internal fixation	342 (70.81)	60 (17.54)

### Thrombosis incidence

As shown in Table 1, the overall incidence of PVT in this study was 18.84%. The incidence was 15.24% in the young/middle-aged group, while it was 23.36% in the elderly group. Additionally, the PVT incidence was 18.32% for males and 19.46% for females. We analysed the incidence of PVT in relation to different fracture sites and types of surgery, and found that the incidence of thrombosis was highest after patellar and knee joint fractures, at 26.09%. This was followed by hip and pelvic fractures at 15.79%, and femoral fractures at 12.45%. Although external fixation was the least common surgical type, it had the highest incidence of PVT at 33.33%. Correlation analysis revealed that age, preoperative coronary disease, duration of surgery and postoperative anticoagulant therapy were significantly associated with the occurrence of PVT. Surprisingly, fracture site and type of surgery did not show a significant association (Table 2).

**Table 2.** Correlation analysis of perioperative factors with postoperative thrombosis at First Affiliated Hospital of Xi’an Jiaotong University and Xi’an Honghui Hospital, China, 2020–2022.

	Correlation coefficient	*p*
Clinically relevant factors		
Age	0.104	0.022*
Sex	0.014	0.751
The site of the fracture	0.076	0.103
Surgery-related factors		
Type of surgery	0.068	0.324
Duration of surgery	0.629	0.000*
Other relevant factors		
Preoperative anticoagulation	0.088	0.054
Postoperative anticoagulation	0.149	0.001*
Preoperative comorbidities		
Coronary disease	0.122	0.007*
Diabetes	0.060	0.191
Hypertension	−0.026	0.562

* *p* < 0.05.

To further investigate the impact of perioperative factors on PVT, we categorized the study population into young/middle-aged groups and elderly groups based on the significant differences in thrombosis incidence observed in different age cohorts. The basic characteristics of the two groups are detailed in Supplemental Table 2.

### Analysis of factors influencing PVT in the young/middle-aged population

In the young/middle-aged population, initial univariate analysis identified 25 factors with statistical significance (Supplemental Table 3), which were subsequently included in multivariate adjustment analysis. The final analysis revealed that the infusion rate of colloids, preoperative prothrombin time activity (PTA) and postoperative anticoagulation significantly influenced the incidence of postoperative thrombosis in this group. Specifically, the infusion rate of colloids (OR = 0.784 (95% CI: 0.667–0.921)) and preoperative PTA (OR = 0.947 (95% CI: 0.913–0.982)) were identified as independent protective factors. Notably, postoperative anticoagulation (OR = 7.117 (95% CI: 2.062–24.560)) was found to be an independent risk factor in this study (Table 3).

**Table 3.** Perioperative predictors of postoperative thrombosis in young/middle-aged patients with lower limb fractures at First Affiliated Hospital of Xi’an Jiaotong University and Xi’an Honghui Hospital, China, 2020–2022.

	OR	95% CI	*p*
The infusion rate of colloids (mL/min)	0.784	0.667–0.921	0.003*
Preoperative prothrombin time activity	0.947	0.913–0.982	0.003^*^
Postoperative anticoagulation	7.117	2.062–24.560	0.002*
Constant	11.761		0.169

* *p* < 0.05.

### Analysis of factors influencing PVT in the elderly population

Similarly, we conducted an analysis of perioperative factors in the elderly population. A total of 21 factors with statistical significance were identified and included in further multivariate adjustment analysis (Supplemental Table 4). The results indicated that the total amount of crystalloids during surgery (mL) (OR = 1.001 (95% CI: 1.000–1.001)) and the infusion rate of colloids (mL/min) (OR = 1.101 (95% CI: 1.004–1.208)) were identified as independent risk factors, increasing the risk of PVT in elderly patients. Conversely, postoperative total protein (TP) (OR = 0.937 (95% CI: 0.886–0.990)) and postoperative serum sodium (OR = 0.918 (95% CI: 0.848–0.994)) were identified as independent protective factors, reducing the risk of PVT in elderly patients (Table 4).

**Table 4.** Perioperative predictors of postoperative thrombosis in elderly patients with lower limb fractures at First Affiliated Hospital of Xi’an Jiaotong University and Xi’an Honghui Hospital, China, 2020–2022.

	OR	95% CI	*p*
Total amount of crystalloids during surgery (mL)	1.001	1.000–1.001	0.007*
Infusion rate of colloids (mL/min)	1.101	1.004–1.208	0.042*
Postoperative total protein	0.937	0.886–0.990	0.020*
Postoperative serum sodium	0.918	0.848–0.994	0.036*
Constant			0.022*

* *p* < 0.05.

### Development of a prediction model for postoperative thrombosis

**Figure 2.**
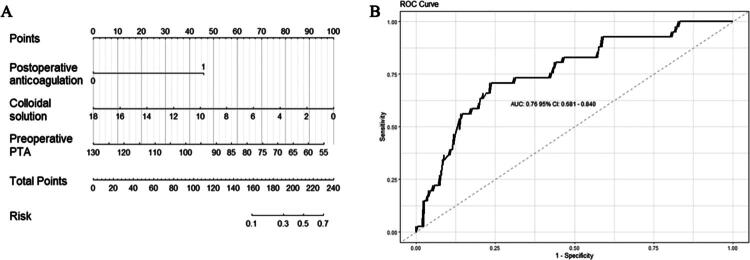
The nomogram predictive model for the young/middle-aged population. (A) The nomogram for the young/middle-aged population; (B) ROC curves for the young/middle-aged population.

Based on the results of the multivariate analysis, we developed nomogram prediction models for each group. The nomogram for the young/middle-aged population is shown in Figure 2(A). The model has an AUC of 0.760 (95% CI: 0.681–0.840) with the cutoff score of 0.465, the sensitivity of 70.7% and specificity of 75.8% (Figure 2(B)). In the nomogram, a total score of 204 or above indicates a significantly increased risk of PVT. Internal validation via 500 bootstrap resampling yielded a *C*-index of 0.754 (95% CI: 0.752–0.763), indicating high calibration of the predictive nomogram. The Brier score was 0.114, reflecting good predictive performance of the model.

**Figure 3.**
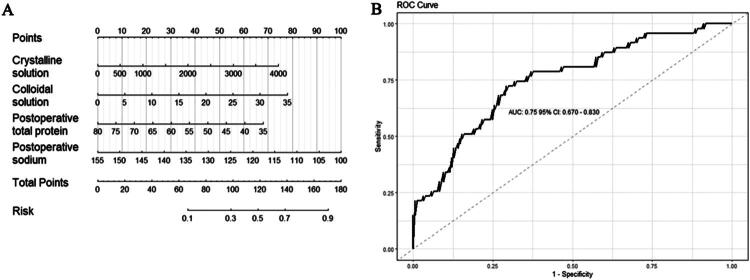
The nomogram predictive model for the elderly population. (A) The nomogram for the elderly population; (B) ROC curves for the elderly population.

The nomogram for the elderly population is depicted in Figure 3(A). This model has an AUC of 0.750 (95% CI: 0.670–0.830), with a sensitivity of 72.3% and specificity of 70.7% (Figure 3(B)). The cutoff value for this model is 0.430, and a score of 116 or above in the nomogram signifies a significantly higher risk of PVT in elderly patients. The model’s internal validation *C*-index is 0.740 (95% CI: 0.737–0.753), indicating high calibration. With a Brier score of 0.146, the model demonstrates good predictive performance.

## Discussion

Thrombosis is a common postoperative complication following fractures and is a major factor limiting the recovery of patients with lower limb fractures [14], as well as increasing mortality rates [15]. Our study revealed a PVT incidence of 18.84%, demonstrating a significant correlation with age, with the elderly population exhibiting a notably higher PVT incidence of 23.36%. Previous studies have seldom conducted independent analyses across different age strata. Through stratified analysis, this study identified distinctly different influencing factors for PVT across age groups. Specifically, preoperative INR, intraoperative colloid infusion volume and postoperative anticoagulation were significant factors in young/middle-aged patients, whereas intraoperative crystalloid and colloid infusion volumes, along with postoperative TP and sodium levels, were critical determinants in the elderly population.

Current perioperative fluid management emphasizes optimizing fluid therapy and volume expansion using blood substitutes such as colloid or saline solutions [16], although there is no evidence that fluid optimization strategies improve patient outcomes [17–19]. In our study, fluid management yielded divergent outcomes between the two populations. The intraoperative infusion rate of colloids was identified as a protective factor for the young/middle-aged population, whereas the total amount of crystalloids and the infusion rate of colloids were risk factors for the elderly. Fracture patients often experience dehydration and blood loss due to trauma, anaesthesia and surgery, increasing risks of traumatic and perioperative complications, particularly in the elderly, making appropriate fluid resuscitation critical [20]. On one hand, inadequate fluid resuscitation may reduce tissue perfusion and oxygen delivery, potentially leading to cellular dysfunction, organ damage or failure, and even death. On the other hand, fluid overload can also be detrimental, causing extreme rightward shift in the Starling myocardial performance curve, respiratory failure due to pulmonary fluid accumulation, gastrointestinal dysmotility and poor wound healing, which may further exacerbate cardiac function [21]. For young/middle-aged patients, stable blood volume helps maintain hemodynamic stability and reduces abnormal platelet aggregation on vascular walls [22]. Furthermore, colloids improve microcirculation and tissue perfusion by expanding blood volume and regulating plasma oncotic pressure; rapid colloid infusion not only improves microcirculation but also minimizes coagulation factor deposition and platelet aggregation, further lowering thrombosis risk [23,24]. However, in elderly patients, diminished physiological regulatory capacity disrupts the dynamic balance between coagulation and anticoagulation systems, predisposing them to hypercoagulability [25,26]. Additionally, excessive or rapid fluid administration may abruptly increase circulatory volume, elevating cardiac preload. Crystalloids may induce vascular endothelial injury, activating the extrinsic coagulation pathway and disrupting coagulation – fibrinolysis balance, while rapid colloid infusion may further aggravate hypercoagulability [27,28]. Therefore, perioperative fluid management requires personalized protocols tailored to individual physiological profiles to mitigate postoperative complications and enhance recovery.

Hypercoagulability is a primary driver of thrombosis formation [29]. In this study, preoperative PTA was identified as a protective factor for the young/middle-aged population. While PTA reflects similar information to prothrombin time (PT), it serves as a more precise indicator of coagulation factor activity. Substantial haemostatic activation is an immediate response to trauma [30], and elevated PTA preoperatively may indicate a better prognosis for young/middle-aged patients. It is generally considered that almost all patients undergoing fracture surgery are at high risk for PVT and are advised to receive preventive interventions, including physical therapy and pharmacotherapy [31]. However, unexpectedly, postoperative anticoagulation therapy was found to be a risk factor for the young/middle-aged population, and it did not provide beneficial effects for the elderly population. In fact, guidelines do not recommend chemical prophylaxis for patients with isolated lower limb fractures and no other risk factors (level of evidence: moderate) [32]. This study further confirms that anticoagulation provides no benefit to younger populations, potentially due to disruption of their intrinsic coagulation–anticoagulation balance. Notably, multiple studies have found no reduction in postoperative DVT incidence with prophylactic anticoagulation [33–35]. Current guidelines primarily recommend anticoagulation for major orthopaedic surgeries [6]. Our inclusion of patients undergoing minor-to-moderate procedures may partially explain these findings, as even the elderly group derived no clinical benefit. In conclusion, the decision to implement postoperative anticoagulation for lower limb fractures requires comprehensive evaluation of age, comorbidities, clinical symptoms and coagulation profiles, with dynamic adjustment of regimens. Routine postoperative anticoagulation may lack specificity and could inadvertently increase risks.

Multiple biomarkers have been linked to a higher risk of VTE [36]. Among the elderly, postoperative TP and serum sodium were identified as protective factors. TP reduces the risk of thrombosis through multiple mechanisms, including protecting vascular endothelial function, regulating the coagulation–anticoagulation balance, optimizing haemorheological properties, exerting antioxidant and anti-inflammatory effects, promoting tissue repair, and clearing metabolic waste [37–44]. Serum sodium also mitigates thrombosis risk in several ways. First, optimal sodium levels maintain blood fluidity, enhancing blood flow and reducing the risk of thrombosis. Second, by stabilizing blood pressure and alleviating vascular wall stress, sodium helps preserve endothelial integrity. Additionally, sodium regulates the dynamic equilibrium between procoagulant and anticoagulant systems [45–48]. Sodium imbalance, whether hypernatremia or hyponatremia, is associated with an increased risk of VTE or mortality [11]. Therefore, maintaining normal serum sodium levels is crucial for elderly patients. For elderly patients, we recommend routine monitoring of postoperative TP and serum sodium levels.

The final prediction models established in this study demonstrated excellent performance, with the young/middle-aged group model achieving an AUC of 0.760, a sensitivity of 70.7%, and a specificity of 75.8%, while the elderly group model attained an AUC of 0.750, a sensitivity of 72.3%, and a specificity of 70.7%. Both models showed outstanding discrimination and calibration capabilities, indicating substantial clinical application value and promising potential for widespread implementation in future clinical practice.

When consulting on the risks and benefits of surgical intervention for fracture patients, surgeons should thoroughly assess the patient’s overall health status to provide personalized treatment recommendations. Additionally, careful consideration should be given to the selection of perioperative fluid management and anticoagulation therapy measures. For elderly patients, particular attention should be paid to postoperative nutritional status to reduce the risk of thrombosis and promote rapid recovery. Through these measures, we can better manage the treatment of fracture patients and reduce the incidence of postoperative thrombosis. This study was conducted at two representative orthopaedic research centres in Northwest China, a region with a population of 103.52 million. The enrolled cohort demonstrates regional representativeness for Asian populations, enhancing the generalizability of findings. However, this study did not conduct subgroup analyses. Future studies could perform further analyses based on factors such as different surgical types and fracture sites.

## Conclusions

In summary, this study identified predictors of DVT for both the young/middle-aged and elderly populations, and constructed corresponding predictive models with robust predictive efficacy, offering valuable guidance for clinical management and treatment. For the young/middle-aged patients, postoperative anticoagulation should be used cautiously. For elderly patients, intraoperative fluid volume and infusion rate must be strictly controlled. It is recommended to closely monitor PTA, TP and serum sodium levels within 24 h postoperatively in fracture patients. Importantly, tailored perioperative fluid management should be implemented for distinct patient populations.

The study population was drawn from two influential centres in Northwest China, making the findings particularly relevant to Asian populations. Performing age-stratified analyses for young/middle-aged and elderly patients separately, we developed highly precise prediction models with strong clinical applicability. However, absence of African, American and other populations limits the generalizability of the results. Additionally, the reported incidence rates covered only the first seven days postoperatively, allowing for an assessment of immediate postoperative complications but lacks long-term follow-up data (including functional outcomes), which limits our comprehensive understanding of patients’ long-term recovery. Future research should consider a broader population and extended follow-up to evaluate the impact of surgery more comprehensively on patients’ long-term quality of life.

## Supplementary Material

Revised Supplemental Digital Content.docx

## Data Availability

The data that support the findings of this study are available from the corresponding author, Xin Shen, upon reasonable request.
