# Supplementary material for: Incidence and factors influencing the occurrence of postoperative thrombosis in patients with lower limb fractures across different age groups: a retrospective study
Source: Ann Med. 2026 Aug 9;58(1):2585602. doi: 10.1080/07853890.2025.2585602 (PMC13459317; doi:10.1080/07853890.2025.2585602)
Supplement: Supplementary file 1 [file IANN_A_2585602_SM5542.docx]

**Article title:**

Incidence and Factors Influencing the Occurrence of Postoperative Thrombosis in Patients with Lower Limb Fractures Across Different Age Groups: A retrospective study

**Supplemental:**

Supplemental Table 1 Study Variables

| Preoperative indicators | age, sex, height, weight, BMI, comorbidity, anticoagulation, the site of the fracture, prothrombin time, prothrombin time activity, international normalized prothrombin ratio, activated partial thromboplastin time, thrombin time, fibrinogen, d-dimer, white blood cell count, neutrophil count, red blood cell count, hemoglobin, platelet count, alanine aminotransferase, aspartate aminotransferase, total bilirubin, direct bilirubin, indirect bilirubin, total protein, albumin, blood urea nitrogen, blood creatinine, potassium, sodium, glucose |
| --- | --- |
| Intraoperative indicators | type of surgery, duration of surgery, crystals, colloids, red blood cells, plasma, total input, urine output, blood loss, total output, fluid balance |
| Postoperative indicators | anticoagulation, PVT, prothrombin time, prothrombin time activity, international normalized prothrombin ratio, activated partial thromboplastin time, thrombin time, fibrinogen, d-dimer, white blood cell count, neutrophil count, red blood cell count, hemoglobin, platelet count, alanine aminotransferase, aspartate aminotransferase, total bilirubin, direct bilirubin, indirect bilirubin, total protein, albumin, blood urea nitrogen, blood creatinine, potassium, sodium, glucose |

Supplemental Table 2 Demographic and Clinical Factors, Biochemical Indicators, Intraoperative Indicators and Postoperative Indicators of Study Group with Lower Limb Fractures at First Affiliated Hospital of Xi'an Jiaotong University and Xi'an Honghui Hospital, China, 2020-2022

|  | Young/middle - aged group (n=269) | Elderly group (n=214) | *p* |
| --- | --- | --- | --- |
| Preoperative indicators |  |  |  |
| Age (y), Median (IQR) | 41 (31,51) | 75 (68,83) | **0.018*** |
| Sex, N (%) |  |  | 0.751 |
| Male | 179 (66.50) | 83 (38.80) |  |
| Female | 90 (33.50) | 131 (61.20) |  |
| Height (m), Median (IQR) | 1.70 (1.62, 1.75) | 1.61 (1.58, 1.70) | 0.499 |
| Weight (kg), Median (IQR) | 68.0 (60.0, 75.0) | 60.0 (52.0, 68.0) | 0.871 |
| BMI (kg/m^2^), Median (IQR) | 23.88 (21.81, 26.09) | 22.05 (20.00, 24.49) | 0.955 |
| Comorbidity, N (%) |  |  |  |
| Hypertension | 21 (7.80) | 80 (37.40) | 0.562 |
| Diabetes | 23 (8.60) | 51 (23.80) | 0.190 |
| Cardiovascular disease | 16 (5.90) | 70 (32.70) | **0.008*** |
| Anticoagulation, N (%) |  |  | 0.054 |
| Yes | 182 (67.70) | 143 (66.80) |  |
| No | 87 (32.30) | 71 (33.20) |  |
| Biochemical indicators, Median (IQR) |  | | |
| Prothrombin time | 12.50 (11.80, 13.40) | 12.55 (11.68, 13.33) | 0.065 |
| Prothrombin time activity | 95.95 (89.13, 100.28) | 93.10 (87.30,1 00.60) | 0.052 |
| International normalized prothrombin ratio | 1.04 (1.00, 1.10) | 1.06 (1.00, 1.11) | **0.034*** |
| Activated partial thromboplastin time | 28.40 (26.20, 31.95) | 28.50 (26.00, 32.35) | 0.650 |
| Thrombin time | 16.60 (15.90, 17.50) | 17.00 (16.10, 17.90) | **0.023*** |
| Fibrinogen | 3.66 (2.78, 4.72) | 3.87 (3.11, 4.64) | 0.790 |
| D-dimer | 1.51 (0.89, 2.79) | 2.51 (1.36, 4.63) | 0.708 |
| White blood cell count | 7.45 (5.97, 9.35) | 6.86 (5.42, 8.59) | 0.655 |
| Neutrophil count | 5.29 (3.98, 7.04) | 5.06 (3.76, 6.81) | 0.540 |
| Red blood cell count | 3.97 (3.55, 4.45) | 3.49 (3.19, 3.91) | 0.529 |
| Hemoglobin | 120.0 (106.00, 135.00) | 106.00 (95.00, 119.25) | 0.771 |
| Platelet count | 228.00 (175.00, 292.50) | 201.00 (157.50, 255.50) | 0.552 |
| Alanine aminotransferase | 25.00 (16.30, 39.80) | 16.20 (11.75, 24.25) | 0.086 |
| Aspartate aminotransferase | 24.00 (17.00, 33.80) | 19.60 (16.05, 24.30) | 0.323 |
| Total bilirubin | 14.70 (11.10, 21.15) | 14.50 (10.00, 19.40) | 0.247 |
| Direct bilirubin | 6.10 (4.40, 8.40) | 5.20 (3.60, 7.10) | 0.219 |
| Indirect bilirubin | 8.70 (6.10, 12.90) | 8.80 (6.00, 12.80) | **0.020*** |
| Total protein | 63.50 (59.10, 67.80) | 60.40 (56.20, 65.50) | 0.500 |
| Albumin | 39.85 (35.90, 43.30) | 35.55 (31.38, 39.23) | 0.371 |
| Blood urea nitrogen | 4.70 (3.80, 5.69) | 5.80 (4.63, 7.69) | 0.655 |
| Blood creatinine | 56.00 (46.00, 68.00) | 59.00 (48.00, 70.75) | 0.353 |
| Potassium | 3.96 (3.70, 4.23) | 3.97 (3.68, 4.32) | 0.872 |
| Sodium | 139.60 (137.93, 141.48) | 140.20 (137.10, 142.80) | 0.994 |
| Glucose | 6.27 (5.30, 7.36) | 7.00 (5.70, 8.90) | 0.505 |
| Intraoperative indicators |  | | |
| Duration of surgery (min) | 182.0 (128.0, 258.5) | 125.0 (95.0 ,170.0) | 0.131 |
| Crystals (ml) | 1000 (500, 2000) | 500 (500, 1000) | **0.000*** |
| Crystals (ml/kg) | 18.75 (8.33, 31.25) | 10.21 (8.03, 20.00) | **0.000*** |
| Crystals (ml/min) | 7.14 (4.13, 10.34) | 5.38 (3.33, 10.06) | **0.000*** |
| Colloids (ml) | 500 (500, 1000) | 500 (0, 500) | 0.906 |
| Colloids (ml/kg) | 7.69 (5.00, 12.99) | 7.75 (0.00, 9.43) | 0.724 |
| Colloids (ml/min) | 2.78 (1.18, 4.30) | 3.33 (0.00, 5.00) | 0.997 |
| Red blood cells (ml) | 0 (0, 260) | 0 (0, 260) | **0.000*** |
| Red blood cells (ml/kg) | 0.00 (0.00, 4.23) | 0.00 (0.00, 4.08) | **0.000*** |
| Red blood cells (ml/min) | 0.00 (0.00, 1.22) | 0.00 (0.00, 1.45) | **0.000*** |
| Plasma (ml) | 0 (0, 0) | 0 (0, 0) | **0.001*** |
| Plasma (ml/kg) | 0.00 (0.00, 0.00) | 0.00 (0.00, 0.00) | **0.002*** |
| Plasma (ml/min) | 0.00 (0.00, 0.00) | 0.00 (0.00, 0.00) | **0.002*** |
| Total Input (ml) | 2300 (1760, 3150) | 1600 (1100, 2100) | **0.001*** |
| Total Input (ml/kg) | 35.38 (26.25, 46.15) | 27.69 (21.33, 37.52) | **0.000*** |
| Total Input (ml/min) | 12.63 (9.52, 16.08) | 12.82 (9.04, 17.50) | 0.063 |
| Total Input (ml/kg/min) | 0.19 (0.14, 0.25) | 0.22 (0.15, 0.30) | 0.084 |
| Urine output (ml) | 500 (300, 800) | 400 (200, 600) | 0.263 |
| Blood loss (ml) | 200 (50, 600) | 200 (100, 300) | **0.018*** |
| Total Output (ml) | 800 (430, 1450) | 600 (300, 900) | **0.044*** |
| Total Output (ml/kg) | 12.50 (6.35, 21.17) | 9.88 (5.85, 15.41) | **0.028*** |
| Total Output (ml/min) | 4.55 (2.72, 6.67) | 4.23 (2.80, 6.34) | 0.091 |
| Total Output (ml/kg/min) | 0.07 (0.04, 0.10) | 0.07 (0.05, 0.11) | 0.093 |
| Fluid balance (ml) | 1400 (900, 1900) | 1000 (700, 1400) | **0.015*** |
| Postoperative indicators |  |  |  |
| Anticoagulation, N (%) |  |  | **0.001*** |
| Yes | 195 (72.50) | 152 (71.00) |  |
| No | 74 (27.50) | 62 (29.00) |  |
| Biochemical indicators, Median (IQR) |  |  |  |
| Prothrombin time | 12.80 (12.00, 13.70) | 12.60 (11.70, 13.53) | 0.589 |
| Prothrombin time activity | 93.90 (87.30, 98.90) | 92.15 (86.00, 99.70) | 0.527 |
| International normalized prothrombin ratio | 1.06 (1.01, 1.12) | 1.06 (1.01, 1.12) | 0.693 |
| Activated partial thromboplastin time | 28.65 (26.23, 32.20) | 28.30 (25.60, 31.40) | 0.168 |
| Thrombin time | 16.40 (15.73, 17.00) | 16.80 (16.20, 17.50) | 0.907 |
| Fibrinogen | 3.74 (2.99, 4.85) | 3.73 (3.25, 4.59) | 0.369 |
| D-dimer | 1.77 (1.00, 3.23) | 2.43 (1.37, 4.94) | 0.217 |
| White blood cell count | 8.78 (7.06, 10.39) | 7.95 (6.37, 9.61) | 0.908 |
| Red blood cell count | 3.88 (3.48, 4.27) | 3.66 (3.20, 4.01) | **0.007*** |
| Hemoglobin | 117.00 (104.50, 132.00) | 111.00 (100.00, 120.25) | **0.040*** |
| Platelet count | 209.00 (163.50, 258.00) | 178.50 (140.50, 226.50) | 0.688 |
| Neutrophil count | 6.95 (5.41, 8.67) | 6.08 (4.77, 7.85) | 0.503 |
| Alanine aminotransferase | 25.30 (17.00, 39.00) | 17.00 (12.95, 25.25) | 0.978 |
| Aspartate aminotransferase | 25.30 (18.60, 33.90) | 22.00 (17.80, 29.00) | 0.913 |
| Total bilirubin | 16.05 (12.00, 23.05) | 14.90 (10.40, 20.70) | 0.559 |
| Direct bilirubin | 6.70 (4.50, 9.60) | 5.50 (3.80, 8.00) | **0.024*** |
| Indirect bilirubin | 8.90 (6.40, 13.20) | 8.80 (5.80, 12.90) | **0.015*** |
| Total protein | 57.50 (52.70, 61.80) | 58.40 (52.90, 64.25) | **0.000*** |
| Albumin | 35.10 (32.13, 38.15) | 33.70 (30.88, 36.53) | **0.000*** |
| Blood urea nitrogen | 4.40 (3.30, 5.32) | 5.76 (4.36, 7.70) | 0.694 |
| Blood creatinine | 52.00 (42.00, 66.00) | 58.00 (49.00, 68.75) | 0.565 |
| Potassium | 3.96 (3.70, 4.21) | 3.98 (3.65, 4.24) | 0.447 |
| Sodium | 139.10 (136.55, 141.60) | 139.50 (137.00, 142.05) | **0.011*** |

*: *p＜0.05*

Supplemental Table 3 Univariate Analysis of Young/Middle-Aged Population with Lower Limb Fractures at First Affiliated Hospital of Xi'an Jiaotong University and Xi'an Honghui Hospital, China, 2020-2022

|  | Postoperative Venous Thrombosis | |  |
| --- | --- | --- | --- |
|  | No (n=228) | Yes (n=41) | *p* |
| Basic Characteristics |  |  |  |
| Age (y), Median (IQR) | 41 (31, 51) | 43 (34, 53) | 0.269 |
| Sex, N (%) |  |  |  |
| Male | 151 (66.23) | 28 (68.29) |  |
| Female | 77 (33.77) | 13 (31.71) | 0.796 |
| Height (m), Median (IQR) | 1.70 (1.62, 1.75) | 168.56 ± 8.39 | 0.850 |
| Weight (kg), Median (IQR) | 68.00 (60.00, 75.00) | 68.88 ± 13.68 | 0.881 |
| BMI (kg/m^2^), Median (IQR) | 24.02 (21.87, 26.11) | 24.19 ± 4.12 | 0.856 |
| Preoperative indicators |  |  |  |
| Hypertension, N (%) |  |  |  |
| No | 209 (91.67) | 39 (95.12) |  |
| Yes | 19 (8.33) | 2 (4.88) | 0.453 |
| Diabetes, N (%) |  |  |  |
| No | 207 (90.79) | 39 (95.12) |  |
| Yes | 21 (9.21) | 2 (4.88) | 0.369 |
| Cardiovascular disease, N (%) |  |  |  |
| No | 213 (93.42) | 40 (97.56) |  |
| Yes | 15 (6.58) | 1 (2.44) | 0.323 |
| Anticoagulation, N (%) |  |  |  |
| No | 78 (34.21) | 9 (21.95) |  |
| Yes | 150 (65.79) | 32 (78.05) | 0.127 |
| Biochemical indicators, Median (IQR) |  | | |
| Prothrombin time | 12.50 (11.72, 13.30) | 12.80 (12.20, 13.55) | **0.034*** |
| Prothrombin time activity | 95.29 ± 9.48 | 90.42 ± 10.17 | **0.004*** |
| International normalized prothrombin ratio | 1.03 (0.99, 1.09) | 1.09 ± 0.09 | **0.008*** |
| Activated partial thromboplastin time | 28.45 (26.02, 31.85) | 30.44 ± 6.54 | 0.292 |
| Thrombin time | 16.70 (15.90, 17.50) | 16.88 ± 1.71 | 0.657 |
| Fibrinogen | 3.66 (2.78, 4.64) | 3.73 (2.80, 5.18) | 0.395 |
| D-dimer | 1.47 (0.88, 2.88) | 1.56 (0.85, 2.31) | 0.665 |
| White blood cell count | 7.26 (5.95, 9.18) | 8.40 ± 2.73 | 0.135 |
| Neutrophil count | 5.28 (3.94, 6.96) | 5.93 (4.59, 7.42) | 0.141 |
| Red blood cell count | 3.98 (3.55, 4.45) | 3.94 ± 0.65 | 0.619 |
| Hemoglobin | 120.56 ± 22.97 | 119.32 ± 20.73 | 0.746 |
| Platelet count | 226.50 (173.00, 291.00) | 238.00 (190.50, 309.50) | 0.068 |
| Alanine aminotransferase | 25.15 (17.00, 41.55) | 22.00 (12.80, 36.65) | 0.382 |
| Aspartate aminotransferase | 24.00 (17.55, 33.85) | 22.50 (15.55, 35.65) | 0.871 |
| Total bilirubin | 14.70 (11.10, 21.50) | 16.78 ± 9.96 | 0.613 |
| Direct bilirubin | 6.00 (4.28, 8.40) | 7.58 ± 5.39 | 0.382 |
| Indirect bilirubin | 8.75 (6.20, 13.00) | 8.00 (6.00, 11.50) | 0.161 |
| Total protein | 63.51 ± 7.07 | 62.60 ± 7.10 | 0.447 |
| Albumin | 40.10 (36.20, 43.40) | 39.03 ± 5.56 | 0.364 |
| Blood urea nitrogen | 4.70 (3.80, 5.61) | 5.08 ± 1.76 | 0.535 |
| Blood creatinine | 56.00 (46.00, 67.25) | 55.00 (44.00, 70.00) | 0.466 |
| Potassium | 3.99 ± 0.38 | 4.07 ± 0.46 | 0.258 |
| Sodium | 139.60 (138.00, 141.57) | 139.03 ± 3.50 | 0.629 |
| Glucose | 6.30 (5.30, 7.36) | 6.21 (5.37, 7.36) | 0.995 |
| Intraoperative indicators |  | | |
| Duration of surgery (min) | 180.00 (125.00, 245.00) | 205.00 (162.50, 322.50) | **0.010*** |
| Crystals (ml) | 1000.00 (500.00, 2000.00) | 1987.80 ± 1315.71 | **0.002*** |
| Crystals (ml/kg) | 20.59 ± 15.81 | 29.19 ± 18.09 | **0.003*** |
| Crystals (ml/min) | 7.25 ± 4.42 | 8.64 ± 4.59 | 0.068 |
| Colloids (ml) | 500.00 (500.00, 1,000.00) | 500.00 (250.00, 750.00) | 0.432 |
| Colloids (ml/kg) | 7.69 (5.05, 13.33) | 7.69 (2.50, 11.11) | 0.561 |
| Colloids (ml/min) | 3.00 (1.19, 4.52) | 2.20 (0.45, 3.70) | **0.023*** |
| Red blood cells (ml) | 0.00 (0.00, 260.00) | 260.00 (0.00, 520.00) | **0.001*** |
| Red blood cells (ml/kg) | 0.00 (0.00, 3.71) | 4.00 (0.00, 7.43) | **0.002*** |
| Red blood cells (ml/min) | 0.00 (0.00, 1.08) | 1.05 (0.00, 2.85) | **0.007*** |
| Plasma (ml) | 0.00 (0.00, 0.00) | 150.00 (0.00, 200.00) | **0.007*** |
| Plasma (ml/kg) | 0.00 (0.00, 0.00) | 2.00 (0.00, 4.18) | **0.009*** |
| Plasma (ml/min) | 0.00 (0.00, 0.00) | 0.36 (0.00, 1.14) | **0.032*** |
| Total Input (ml) | 2200.00 (1600.00, 2900.00) | 2700.00 (2200.00, 4010.00) | **0.004*** |
| Total Input (ml/kg) | 33.27 (25.10, 43.49) | 40.00 (35.78, 53.93) | **0.000*** |
| Total Input (ml/min) | 13.58 ± 6.37 | 13.00 (11.12, 15.44) | 0.895 |
| Total Input (ml/kg/min) | 0.19 (0.14, 0.25) | 0.20 (0.15, 0.24) | 0.958 |
| Urine output (ml) | 500.00 (300.00, 800.00) | 700.00 (400.00, 1,000.00) | **0.022*** |
| Blood loss (ml) | 200.00 (50.00, 600.00) | 600.00 (100.00, 925.00) | **0.031*** |
| Total Output (ml) | 800.00 (402.50, 1300.00) | 1200.00 (650.00, 2000.00) | **0.009*** |
| Total Output (ml/kg) | 11.76 (6.06, 20.00) | 18.67 (9.75, 32.35) | **0.004*** |
| Total Output (ml/min) | 4.34 (2.52, 6.63) | 5.60 (3.17, 8.04) | 0.080 |
| Total Output (ml/kg/min) | 0.08 ± 0.06 | 0.08 (0.05, 0.12) | 0.117 |
| Fluid balance (ml) | -1400.00 (-1800.00, -900.00) | 1603.41 ± 744.50 | 0.156 |
| Postoperative indicators |  |  |  |
| Anticoagulation, N (%) |  |  |  |
| No | 71 (31.14) | 3 (7.32) |  |
| Yes | 157 (68.86) | 38 (92.68) | **0.005*** |
| Biochemical indicators, Median (IQR) |  |  |  |
| Prothrombin time | 12.80 (11.90, 13.70) | 12.70 (12.15, 13.80) | 0.594 |
| Prothrombin time activity | 93.90 (87.68, 99.52) | 93.90 (85.90, 98.10) | 0.479 |
| International normalized prothrombin ratio | 1.06 (1.01, 1.12) | 1.06 (1.02, 1.14) | 0.903 |
| Activated partial thromboplastin time | 28.60 (26.10, 31.90) | 28.90 (26.90, 32.75) | 0.829 |
| Thrombin time | 16.40 (15.80, 17.10) | 16.22 ± 0.75 | 0.105 |
| Fibrinogen | 3.69 (2.98, 4.82) | 4.12 (3.32, 4.96) | 0.188 |
| D-dimer | 1.74 (0.94, 3.01) | 2.00 (1.25, 3.71) | 0.825 |
| White blood cell count | 8.76 (6.98, 10.32) | 9.13 (7.13, 11.22) | 0.401 |
| Red blood cell count | 3.89 (3.48, 4.28) | 3.82 ± 0.59 | 0.514 |
| Hemoglobin | 117.57 ± 19.88 | 110.87 ± 22.48 | 0.058 |
| Platelet count | 208.00 (158.00, 255.00) | 229.49 ± 83.972 | 0.370 |
| Neutrophil count | 7.00 (5.26, 8.61) | 6.90 (5.98, 9.72) | 0.311 |
| Alanine aminotransferase | 25.25 (17.00, 39.08) | 30.88 ± 22.00 | 0.403 |
| Aspartate aminotransferase | 25.00 (18.48, 33.75) | 28.34 ± 12.88 | 0.403 |
| Total bilirubin | 16.20 (12.00, 23.10) | 15.70 (12.50, 20.85) | 0.590 |
| Direct bilirubin | 6.55 (4.58, 9.30) | 7.50 (4.35, 10.60) | 0.598 |
| Indirect bilirubin | 9.05 (6.60, 13.65) | 8.50 (5.60, 11.75) | 0.133 |
| Total protein | 58.10 (53.38, 62.50) | 54.71 ± 7.00 | **0.022*** |
| Albumin | 35.51 ± 4.96 | 33.42 ± 4.54 | **0.014*** |
| Blood urea nitrogen | 4.28 (3.30, 5.29) | 4.80 (3.65, 6.00) | 0.531 |
| Blood creatinine | 53.00 (42.00, 66.00) | 50.00 (39.50, 67.50) | 0.699 |
| Potassium | 3.96 ± 0.42 | 4.04 ± 0.32 | 0.278 |
| Sodium | 139.10 (136.65, 141.65) | 137.54 ± 7.69 | 0.599 |

*: *p＜0.05*

Supplemental Table 4 Univariate Analysis of Elderly Population with Lower Limb Fractures at First Affiliated Hospital of Xi'an Jiaotong University and Xi'an Honghui Hospital, China, 2020-2022

|  | Postoperative Venous Thrombosis | |  |
| --- | --- | --- | --- |
|  | No (n=164) | Yes (n=50) | *p* |
| Basic Characteristics |  |  |  |
| Age (y), Median (IQR) | 75 (68, 82) | 74.98 ± 9.065 | 0.805 |
| Sex, N (%) |  |  |  |
| Male | 63 (38.41) | 20 (40.00) |  |
| Female | 101 (61.59) | 30 (60.00) | 0.840 |
| Height (m), Median (IQR) | 1.62 (1.58, 1.70) | 163.50 ± 7.80 | 0.698 |
| Weight (kg), Median (IQR) | 60.00 (52.00, 66.75) | 61.630 ± 12.97 | 0.391 |
| BMI (kg/m^2^), Median (IQR) | 22.04 (20.00, 24.22) | 22.93 ± 3.80 | 0.454 |
| Preoperative indicators |  |  |  |
| Hypertension, N (%) |  |  |  |
| No | 99 (60.37) | 35 (70.00) |  |
| Yes | 65 (39.63) | 15 (30.00) | 0.220 |
| Diabetes, N (%) |  |  |  |
| No | 129 (78.66) | 34 (68.00) |  |
| Yes | 35 (21.34) | 16 (32.00) | 0.124 |
| Cardiovascular disease, N (%) |  |  |  |
| No | 118 (71.95) | 26 (52.00) |  |
| Yes | 46 (28.05) | 24 (48.00) | **0.009*** |
| Anticoagulation, N (%) |  |  |  |
| No | 58 (35.37) | 13 (26.00) |  |
| Yes | 106 (64.63) | 37 (74.00) | 0.220 |
| Biochemical indicators, Median (IQR) |  | | |
| Prothrombin time | 12.55 (11.50, 13.38) | 12.70 ± 1.05 | 0.853 |
| Prothrombin time activity | 93.10 (86.10, 100.98) | 93.96 ± 8.70 | 0.946 |
| International normalized prothrombin ratio | 1.06 (1.00, 1.11) | 3.06 ± 14.13 | 0.403 |
| Activated partial thromboplastin time | 28.75 (26.02, 33.30) | 27.90 (25.52, 31.55) | 0.202 |
| Thrombin time | 16.70 (16.00, 17.70) | 17.55 (16.50, 18.48) | 0.060 |
| Fibrinogen | 4.00 ± 1.3 | 3.60 (3.07, 4.26) | 0.314 |
| D-dimer | 2.55 (1.36, 4.66) | 2.46 (1.32, 4.50) | 0.772 |
| White blood cell count | 7.32 ± 2.71 | 6.96 ± 1.94 | 0.374 |
| Neutrophil count | 5.52 ± 2.30 | 5.24 ± 1.93 | 0.435 |
| Red blood cell count | 3.51±0.56 | 3.56 ± 0.51 | 0.549 |
| Hemoglobin | 105.15 ± 18.78 | 111.80 ± 15.82 | **0.024*** |
| Platelet count | 201.00 (158.00, 265.75) | 198.82±73.40 | 0.209 |
| Alanine aminotransferase | 17.00 (12.00, 24.50) | 15.50 (11.50, 24.42) | 0.463 |
| Aspartate aminotransferase | 19.90 (16.00, 24.10) | 19.00 (16.00, 24.70) | 0.161 |
| Total bilirubin | 14.55 (10.15, 20.70) | 14.39 ± 6.04 | 0.114 |
| Direct bilirubin | 5.15 (3.40, 7.10) | 6.07 ± 2.34 | 0.759 |
| Indirect bilirubin | 8.95 (6.20, 13.42) | 8.20 (5.75, 10.40) | **0.028*** |
| Total protein | 60.42 ± 7.82 | 61.12 ± 6.69 | 0.569 |
| Albumin | 35.05 (30.82, 38.38) | 37.52 ± 5.40 | **0.003*** |
| Blood urea nitrogen | 5.81 (4.70, 7.83) | 5.66 (4.48, 7.02) | 0.091 |
| Blood creatinine | 59.00 (47.75, 70.25) | 60.00 (48.00, 71.75) | 0.604 |
| Potassium | 3.98 (3.69, 4.38) | 3.97 ± 0.46 | 0.402 |
| Sodium | 140.30 (137.10, 142.70) | 140.11 ± 3.791 | 0.562 |
| Glucose | 7.20 (5.84, 8.91) | 6.04 (5.50, 9.01) | 0.372 |
| Intraoperative indicators |  | | |
| Duration of surgery (min) | 122.50 (95.00, 159.75) | 130.00 (98.50, 180.00) | 0.398 |
| Crystals (ml) | 500.00 (500.00, 1,000.00) | 1166.00 ± 796.83 | **0.000*** |
| Crystals (ml/kg) | 9.71 (7.69, 16.67) | 19.10 ± 12.19 | **0.000*** |
| Crystals (ml/min) | 4.60 (3.19, 8.61) | 9.64 ± 6.73 | **0.005*** |
| Colloids (ml) | 500.00 (0.00, 500.00) | 500.00 (500.00, 500.00) | **0.040*** |
| Colloids (ml/kg) | 7.69 (0.00, 9.26) | 8.33 (6.64, 10.34) | 0.095 |
| Colloids (ml/min) | 3.33 (0.00, 4.55) | 4.17 (2.10, 5.28) | **0.037*** |
| Red blood cells (ml) | 0.00 (0.00, 260.00) | 0.00 (0.00, 260.00) | **0.029*** |
| Red blood cells (ml/kg) | 0.00 (0.00, 3.96) | 0.00 (0.00, 4.73) | 0.086 |
| Red blood cells (ml/min) | 0.00 (0.00, 1.42) | 0.00 (0.00, 2.19) | **0.039*** |
| Plasma (ml) | 0.00 (0.00, 0.00) | 0.00 (0.00, 0.00) | 0.355 |
| Plasma (ml/kg) | 0.00 (0.00, 0.00) | 0.00 (0.00, 0.00) | 0.777 |
| Plasma (ml/min) | 0.00 (0.00, 0.00) | 0.00 (0.00, 0.00) | 0.256 |
| Total Input (ml) | 1600.00 (1100.00, 1990.00) | 1800.00 (1470.00, 2650.00) | **0.004*** |
| Total Input (ml/kg) | 26.67 (20.81, 35.77) | 33.53 ± 13.05 | **0.033*** |
| Total Input (ml/min) | 12.50 (8.44, 17.29) | 18.13 ± 20.04 | 0.060 |
| Total Input (ml/kg/min) | 0.22 (0.14, 0.30) | 0.31 ± 0.37 | 0.096 |
| Urine output (ml) | 400.00 (200.00, 600.00) | 400.00 (200.00, 600.00) | 0.946 |
| Blood loss (ml) | 200.00 (100.00, 300.00) | 200.00 (100.00, 400.00) | **0.049*** |
| Total Output (ml) | 600.00 (300.00, 900.00) | 550.00 (375.00, 1012.50) | 0.191 |
| Total Output (ml/kg) | 9.45 (5.91, 14.89) | 12.06 ± 8.80 | 0.439 |
| Total Output (ml/min) | 4.19 (2.81, 6.14) | 5.85 ± 5.86 | 0.118 |
| Total Output (ml/kg/min) | 0.07 (0.04, 0.11) | 0.10 ± 0.11 | 0.203 |
| Fluid balance (ml) | -1000.00 (-1267.50, -700.00) | 1301.60 ± 654.486 | **0.003*** |
| Postoperative indicators |  |  |  |
| Anticoagulation, N (%) |  |  |  |
| No | 52 (31.71) | 10 (20.00) |  |
| Yes | 112 (68.29) | 40 (80.00) | 0.114 |
| Biochemical indicators, Median (IQR) |  |  |  |
| Prothrombin time | 12.50 (11.52, 13.80) | 12.69 ± 0.98 | 0.602 |
| Prothrombin time activity | 92.30 (85.08, 100.82) | 92.85 ± 7.17 | 0.622 |
| International normalized prothrombin ratio | 1.06 (1.01, 1.13) | 1.06 ± 0.06 | 0.314 |
| Activated partial thromboplastin time | 28.50 (26.00, 31.70) | 27.10 (24.88, 29.48) | 0.035 |
| Thrombin time | 16.75 (16.20, 17.50) | 17.02 ± 1.19 | 0.696 |
| Fibrinogen | 3.98 ± 1.18 | 3.70 (3.31, 4.39) | 0.638 |
| D-dimer | 2.43 (1.36, 5.58) | 2.52 (1.40, 3.79) | 0.401 |
| White blood cell count | 8.04 ± 2.56 | 8.08 (6.29, 9.71) | 0.883 |
| Red blood cell count | 3.67 ± 0.60 | 3.50 ± 0.49 | 0.081 |
| Hemoglobin | 111.31 ± 18.64 | 109.90 ± 15.07 | 0.624 |
| Platelet count | 191.26 ± 75.06 | 174.48 ± 76.68 | 0.170 |
| Neutrophil count | 6.38 ± 2.40 | 6.54 (4.80, 8.20) | 0.798 |
| Alanine aminotransferase | 16.00 (12.00, 25.00) | 20.45 (14.22, 29.65) | 0.680 |
| Aspartate aminotransferase | 22.00 (17.50, 29.00) | 22.00 (18.08, 29.95) | 0.785 |
| Total bilirubin | 14.95 (10.30, 20.88) | 14.20 (10.80, 19.00) | 0.530 |
| Direct bilirubin | 4.90 (3.60, 7.90) | 6.60 (5.30, 9.15) | 0.738 |
| Indirect bilirubin | 9.20 (5.98, 13.30) | 6.70 (4.60, 11.60) | 0.067 |
| Total protein | 59.65 ± 7.09 | 54.99 ± 6.63 | **0.000*** |
| Albumin | 34.03 ± 4.48 | 32.29 ± 4.30 | **0.018*** |
| Blood urea nitrogen | 6.14 (4.55, 7.94) | 5.30 (3.72, 6.60) | 0.058 |
| Blood creatinine | 59.00 (49.75, 70.25) | 55.00 (48.75, 66.25) | 0.374 |
| Potassium | 3.97 ± 0.53 | 3.95 ± 0.40 | 0.783 |
| Sodium | 139.88 (137.95, 142.20) | 137.70 (135.70, 140.90) | **0.004*** |

*: *p＜0.05*
